# Focus group in Multiple Sclerosis as a tool to increase active patient involvement. A preliminary experience

**DOI:** 10.1186/1745-6215-16-S1-P9

**Published:** 2015-05-29

**Authors:** Annalisa Sgoifo, A Bignamini, M Celani, L La Mantia, M Esposito, R Marazzi, V Prone, A Protti, V Sangalli, E Agostoni

**Affiliations:** 1 Interventionalist Neuroradiology Department, Niguarda Ca' Granda Hospital, Milan - Italy; 2 Department of Pharmaceutical Sciences (DISFARM) - University of Milan, Milan - Italy; 3 UOC Neurophysiopathology - A.O. Perugia, Perugia - Italy; 4 Neurology and Stroke Unit, Neurosciences Department - Niguarda Ca’ Granda Hospital, Milan – Italy

## Background

Unmet needs may influence emotional status, life style and care [1]. Our aims were to involve persons with Multiple Sclerosis (pwMS) to actively take care of themselves and disclose patient-centered outcome measures [2].

## Materials and methods

Focus group was organized at the end of an educational MS Open-Day meeting at Niguarda Hospital. Focus group (FG) was explained through personal contact, at entry, and by a brief oral presentation. Participating pwMS filled out a registration form, declaring their consent to participate. Two questions were selected. FG was organized by expert psychotherapist and conducted by two psychotherapists. Conversation was recorded, computerized and examined anonymously, using qualitative and quantitative text analysis.

## Results

25 registration form were distributed, 12 subscriptions were collected and six pwMS participated. PwMS (5 women and one man) were regularly followed by MS Centre. Mean age was 50 (range 26-81); EDSS mean was 2.6 (range 1-6.5). Five patients were affected by relapsing remitting and one by secondary progressive MS. Disease duration ranged from 2 up to 34 years. Group discussion lasted 72 minutes. Two main questions were delivered: A) what did you wish for yourself? B) what do you consider most important to improve MS care? The replies were the following: A) All patients described an emotional reaction (hate, fear, anger). All declared a strong wish to continue with their life and avoid disability. B) All underlined the importance of: clear information, empathic care, multidisciplinary approach to the disease. The preliminary text analysis showed that patients had an emotional active fighting and not depressive reaction. This reveals an active coping. Main expressed needs were to find strategy to appear normal person in the social setting and to have a referent point (mainly the referent neurologist) to show and express themselves as fragile and affected people.

## Discussion

Data are preliminary for a larger project and limited by small size and selection of participants. FG may facilitate the relationship with clinicians and guide research.

## Conclusion

In our experience FG appears an intriguing technique to investigate patient’s needs, actively involving them in care and research. Main conclusion is that MS was accepted and perceived as a part of life. Decentring their disease, they clearly revealed a better ability to cope with disease focusing on the present, without delaying their needs. The replies were homogenous despite of clinical heterogeneity.
